# Kaposi’s sarcoma: a single-center experience on 38 patients^☆^^☆☆^

**DOI:** 10.1016/j.abd.2021.02.007

**Published:** 2021-07-27

**Authors:** Joana Cruz Matos Calvão da Silva, José Carlos Cardoso, Ricardo Vieira

**Affiliations:** Dermatology Department, Coimbra University Hospital, Coimbra, Portugal

Dear Editor,

Kaposi’s Sarcoma (KS) is a multifocal angioproliferative neoplasm associated with infection by Human Herpes Virus type 8 (HHV8).^1^, ^2^

We retrospectively studied 38 biopsy-proven KS diagnosed at our department between 2010 and 2019.

The patients’ mean age was 60.5 years (range 35–84). The population was mostly male (n = 33, 86.8%) and Caucasian (n = 31, 81.6%).

The epidemic (HIV-associated) subtype predominated (16 cases; 42.1%), followed by the classic (n = 12; 31.6%) and iatrogenic (n = 10; 26.3%), with no endemic cases. The majority of patients (n = 26; 68.4%) were immunocompromised: 16 with HIV infection, seven organs transplanted, and three due to other causes (mainly chronic high dose corticotherapy). The majority (62.5%) of the HIV infected had a CD4 count of <200 cells/mm^3^ (median value of 113). The mean time from transplant to the appearance of KS was 11.9 months (range 5–30).

Cutaneous elementary lesions were papules and/or nodules in 74% of the patients (n = 13), plaques in five, and macules/patches in three (Fig. 1). Almost all (84%) were violaceous. Fifty-two percent had perilesional disease (<10 lesions); eight patients presented with 10–30 lesions and eight with more than 30; in five we could not find this data. Lesions were exclusively located in the lower limbs in 59% (n = 19), followed by the genitals (n = 3), trunk (n = 2), superior extremities (n = 1) and face (n = 1); twelve patients had generalized cutaneous lesions. Extracutaneous involvement occurred in 37% of patients (n = 14), the majority of which (78%) immunosuppressed, especially in the context of HIV (82%). Specifically, mucous membranes were affected in 10 and lymph nodes in five. Visceral involvement was gastrointestinal in all cases (n = 4) and simultaneously pulmonary in three. B symptoms were present in 12.5% (n = 4).

**Fig. 1:**
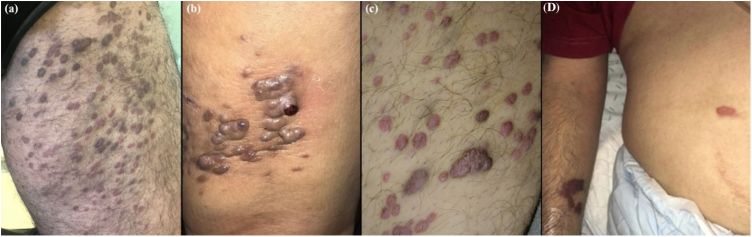
Clinical presentation of Kaposi’s Sarcoma in four patients: (A–C), papulo-nodular, violaceous lesions on the lower extremities (the most common clinical presentation); (D), a small number of violaceous patches in the upper limb and abdomen in an HIV patient.

Histologically, spindle cells (92%), slit-like spaces (84%), extravasated red blood cells (89%), and lymphoplasmacytic infiltrate (73%) were found in almost all cases. Intracellular eosinophilic globules were identified in 34%. Immunohistochemistry for HHV8 was positive in all cases in which it was conducted (n = 14).

A watchful waiting approach was adopted in three cases. When appropriate, reduction and/or change (to mTOR inhibitors) of immunosuppression was done. Local therapies were performed in 12 patients: surgical excision in five, ablative Carbon Dioxide (CO_2_) laser in three, cryotherapy in two, and radiotherapy in two. Systemic therapies were needed as a first-line in the majority of cases (n = 25; 65.8%): Antiretroviral Therapy (ART) in 14 (56%), pegylated liposomal doxorubicin in 8 (32%), bleomycin in 2 (8%) and vinorelbine in 1 (4%). Fourteen patients (36.8%) needed second and/or third-line therapies, with local (laser CO_2_, cryotherapy, radiotherapy, intralesional vinblastine) and/or systemic (doxorubicin, bleomycin, vinorelbine, vinblastine, alfa interferon, paclitaxel) approaches in variable combinations.

Partial and/or complete response was achieved in the majority (65.8%) of cases. A third developed – mainly mild (venous stasis and/or lymphedema) – complications. However, transformation to an anaplastic variant arose in one case (Fig. 2) and another patient was subsequently diagnosed with non-Hodgkin lymphoma. Although overall mortality was 36.8%, mortality directly related to KS was only 8% (n = 3). Two patients had classic KS: one died aged 83 following transformation to anaplastic KS despite four cycles of bleomycin; the other died due to visceral progression of the disease (gastrointestinal and pulmonary involvement already present at the moment of diagnosis, later progressing with liver metastases – Fig. 3). The third patient was heart transplanted, dying with gastrointestinal and pulmonary metastases of KS, even after immunosuppression adjustment and 6 cycles of vinorelbine.

**Fig. 2:**
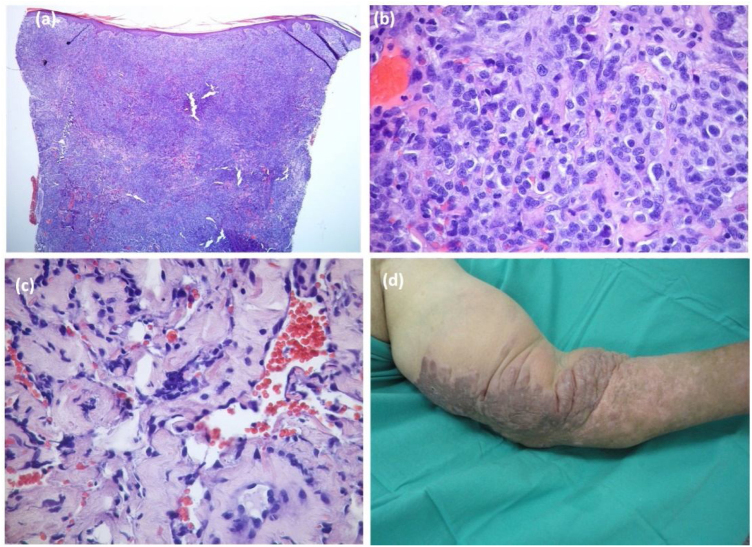
Anaplastic variant of Kaposi’s Sarcoma: histological (A-C), and clinical (D), pictures. Dense tumoral proliferation in the dermis (A, Hematoxylin & eosin ×40), mainly composed of epithelioid cells with pleomorphism and frequent mitoses (B, Hematoxylin & eosin ×400). (C), Dissection of collagen bundles by vascular clefts with atypical endothelial cells (Hematoxylin & eosin ×200). (D) Large and infiltrated violaceous plaque with papillomatous areas in the left upper limb.

**Fig. 3:**
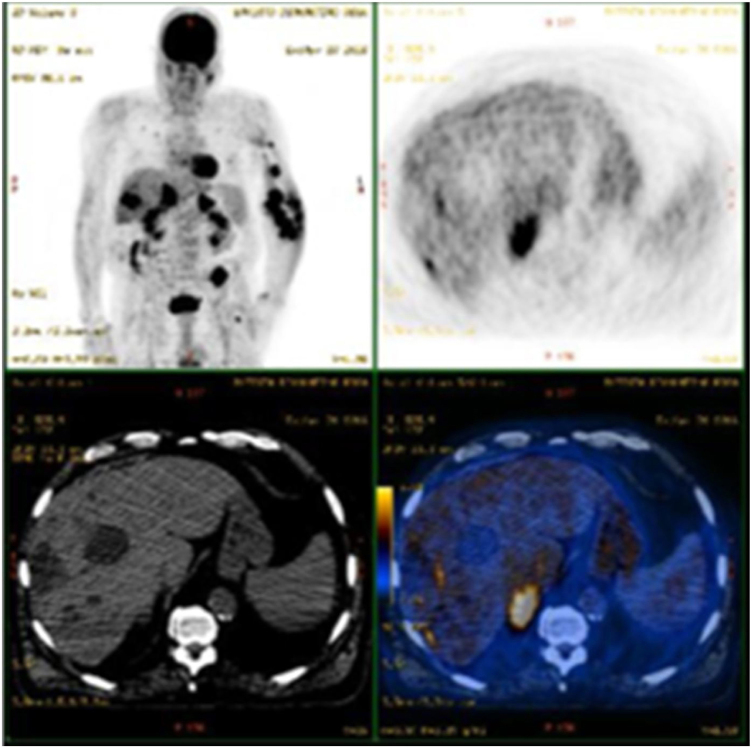
PET-CT scan showing the progression of Kaposi’s Sarcoma with metastatic involvement of the liver in a patient with previously known gastrointestinal and pulmonary involvement. This patient later died despite multiple treatments, including doxorubicin, alfa-interferon, paclitaxel, vinorelbine, and radiotherapy.

We also compared the group of immunocompetent/classic KS patients with immunocompromised ones. Results are summarised in Table 1.

**Table 1:** Comparison between the groups of immunocompetent (classic KS) and immunocompromised patients with KS.

Variable	Classic KS (n = 12)	KS associated with immunosuppression (n = 26)	p-value	Statistical test used
Mean age (years)	73.1	54.5	p < 0.001	Student's *t*-test
Extracutaneous involvement	3	11	p = 0.472	Chi-squared test
Disease-related mortality	2	1	p = 0.229	Chi-squared test

In our study, KS was almost seven times more frequent in men (6.6:1), which is a greater ratio than reported in the literature (2–5:1, at least for the classic variant); ethnicity and age distribution were similar to other European reports.^1^, ^3^, ^4^

A potentially intriguing result is the relatively high percentage of epidemic cases compared to classic KS, which would be expected to predominate in a Caucasian European population like ours. This is likely explained by the fact that the great majority of our KS patients come from the Infectious Disease department. Moreover, our department has a differentiated consultation for immunosuppressed patients, which further explains this specific scenario.

Cutaneous lesions did not differ from what is described in the literature. On the contrary, the percentage of extracutaneous involvement was quite elevated (37%), especially when we compare to other recent studies (15% in a single-Turkish center study published in 2018; and 16.8% in a retrospective study from a tertiary hospital in Barcelona from 1987–2016, which included many HIV patients with advanced disease in the pre-ART era).^3^, ^4^ This may be due to the greater number of immunosuppressed patients in our sample (n = 26, vs. n = 10 classic KS), particularly HIV, which is generally associated with greater extra-cutaneous involvement (as described in the literature and also seen in this study).^1^, ^5^

In accordance with literature data, the prognosis of KS was good: the overall response was observed in 25 cases, stabilization of the disease in two, and progression in four. Disease-specific mortality was 8%, closer to other published studies (e.g. 6.5% in a Turkish study and 5.2% in a Spanish one).^3^, ^4^

Comparing the groups of immunocompetent and immunocompromised patients, the second was significantly younger than the first, which is in accordance with the literature.^6^ As expected, there was a greater prevalence of extracutaneous involvement in immunocompromised patients, although not a statistically significant one (which may be due to the small sample size). There was also no statistical difference in specific mortality by KS.

As far as we know, this is the largest study on KS in the Portuguese population and the first concerning the dermatologic perspective. The study’s main limitations are its retrospective nature and the relatively small sample size.

## Financial support

None declared.

## Authors’ contributions

Joana Cruz Matos Calvão da Silva: Approval of the final version of the manuscript; critical literature review; data collection, analysis, and interpretation; effective participation in research orientation; intellectual participation in propaedeutic and/or therapeutic management of studied cases; manuscript critical review; preparation and writing of the manuscript; statistical analysis; study conception and planning.

José Carlos Cardoso: Approval of the final version of the manuscript; data collection, analysis and interpretation.

Ricardo Vieira: Effective participation in research orientation; intellectual participation in propaedeutic and/or therapeutic management of studied cases; statistical analysis; study conception and planning; approval of the final version of the manuscript.

## Conflicts of interest

None declared.
